# Supplementary material for: Splice-Junction-Based Mapping of Alternative Isoforms in the Human Proteome
Source: Cell Rep. Author manuscript; Available in PMC 2020 Jan 15. (PMC6961840; doi:10.1016/j.celrep.2019.11.026)
Supplement: 3 [file NIHMS1546469-supplement-3.zip › DF2/PXD009737/TestisGluC-225-Q676U5-TSPVRAISRAATRRSVSSFPVPQDNVDTHPGSGKE.pdf]

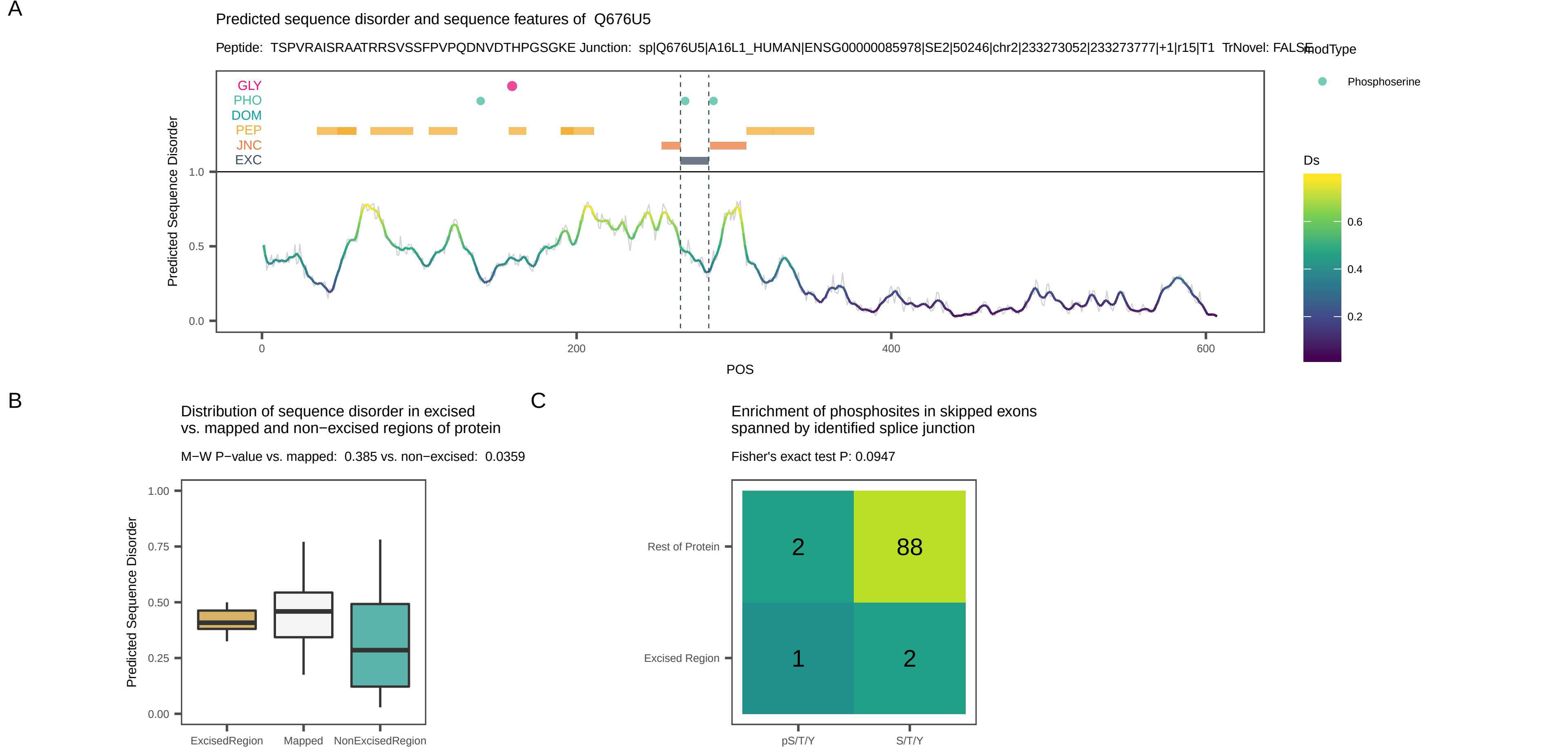

A
Predicted sequence disorder and sequence features of Q676U5
Peptide: TSPVRAISRAATRRSVSSFPVPQDNVDTHPGSGKE Junction: sp|Q676U5|A16L1_HUMAN|ENSG00000085978|SE2|50246|chr2|233273052|233273777|+1|r15|T1 TrNovel: FALSE
modType
Phosphoserine
GLY
PHO
DOM
PEP
JNC
EXC
Predicted Sequence Disorder
1.0
0.5
0.0
0
200
400
600
POS
Ds
0.6
0.4
0.2
B
Distribution of sequence disorder in excised
vs. mapped and non−excised regions of protein
M−W P−value vs. mapped: 0.385 vs. non−excised: 0.0359
Predicted Sequence Disorder
1.00
0.75
0.50
0.25
0.00
ExcisedRegion
Mapped
NonExcisedRegion
C
Enrichment of phosphosites in skipped exons
spanned by identified splice junction
Fisher's exact test P: 0.0947
Rest of Protein
Excised Region
2
88
1
2
pS/T/Y
S/T/Y
